# Digital Health Literacy During the COVID-19 Pandemic Among Health Care Providers in Resource-Limited Settings: Cross-sectional Study

**DOI:** 10.2196/39866

**Published:** 2022-11-14

**Authors:** Mohammedjud Hassen Ahmed, Habtamu Alganeh Guadie, Habtamu Setegn Ngusie, Gizaw Hailiye Teferi, Monika Knudsen Gullslett, Samuel Hailegebreal, Mekonnen Kenate Hunde, Dereje Oljira Donacho, Binyam Tilahun, Shuayib Shemsu Siraj, Gebiso Roba Debele, Mohammedamin Hajure, Shegaw Anagaw Mengiste

**Affiliations:** 1 Department of Health Informatics College of Public Health and Medical Sciences Mettu University Mettu Ethiopia; 2 School of Public Health College of Medicine and Health Sciences Bahir Dar University Bahir Dar Ethiopia; 3 Department of Health Informatics College of Medicine and Health Science Debre Markos University Debre Markos Ethiopia; 4 Faculty of Health and Social Science University of South-East Norway Drammen Norway; 5 Department of Health Informatics College of Medicine and Health Science Arba Minch University Arba Minch Ethiopia; 6 Department of Social Sciences Mettu University Mettu Ethiopia; 7 Department of Health Informatics Mettu University Mettu Ethiopia; 8 Department of Health Informatics University of Gondar Gondar Ethiopia; 9 Department of Public Health Mettu University Mettu Ethiopia; 10 Department of Psychiatry Mettu University Mettu Ethiopia; 11 Department of Management Information Systems University of South East Norway Drammen Norway

**Keywords:** digital, health, literacy, COVID-19, professionals, Ethiopia, health professionals, digital literacy, skills, knowledge, perception, use, education, training

## Abstract

**Background:**

Digital health literacy is the use of information and communication technology to support health and health care. Digital health literacy is becoming increasingly important as individuals continue to seek medical advice from various web-based sources, especially social media, during the pandemics such as COVID-19.

**Objective:**

The study aimed to assess health professionals’ digital health literacy level and associated factors in Southwest Ethiopia in 2021.

**Methods:**

An institution-based cross-sectional study was conducted from January to April 2021 in Ethiopia. Simple random sampling technique was used to select 423 study participants among health professionals. SPSS (version 20) software was used for data entry and analysis. A pretested self-administered questionnaire was used to collect the required data. Multivariable logistic regression was used to examine the association between the digital health literacy skill and associated factors. Significance value was obtained at 95% CI and *P*<.05.

**Results:**

In total, 401 study subjects participated in the study. Overall, 43.6% (n=176) of respondents had high digital health literacy skills. High computer literacy (adjusted odds ratio [AOR] 4.43, 95% CI 2.34-5.67; *P*=.01); master’s degree and above (AOR 3.42, 95% CI 2.31-4.90; *P*=.02); internet use (AOR 4.00, 95% CI 1.78-4.02; *P*=.03); perceived ease of use (AOR 2.65, 95% CI 1.35-4.65; *P*=.04); monthly income of >15,000 Ethiopian birr (>US $283.68; AOR 7.55, 95% CI 6.43-9.44; *P*<.001); good knowledge of eHealth (AOR 2.22, 95% CI 1.32-4.03; *P*=.04); favorable attitudes (AOR 3.11, 95% CI 2.11-4.32; *P*=.04); and perceived usefulness (AOR 3.43, 95% CI 2.43-5.44; *P*=.02) were variables associated with eHealth literacy level.

**Conclusions:**

In general, less than half of the study participants had a high digital health literacy level. High computer literacy, master’s degree and above, frequent internet use, perceived ease to use, income of >15,000 Ethiopian birr (>US $283.68), good knowledge of digital health literacy, favorable attitude, and perceived usefulness were the most determinant factors in the study. Having high computer literacy, frequent use of internet, perceived ease of use, perceived usefulness, favorable attitude, and a high level of education will help to promote a high level of digital health literacy.

## Introduction

Information and communication technologies (ICTs) greatly reduce health disparities through promoting health, preventing disease, and supporting clinical care for all [1-3]. Moreover, the development of ICTs for patient and consumer health apps has been exploding in the past decade, with thousands of websites, hundreds of mobile apps, and dozens of special purpose devices targeted at the health care markets [4-6]. These ICTs enhance the digitalization of health and diversify the use of digital health around the globe; ultimately, it leads to the accessibility of high-quality, cost-effective health care service delivery through improving the communication of health professionals [7-11].

Public health emergencies such as COVID-19 need up-to-date health information for prevention, tackling of the disease, and the protection of the community from long-lasting economic and societal impacts. Digital health is the use of emerging ICT, mainly the internet, to improve health that emphasizes the roles that digital technologies play in facilitating health care, health information delivery and storage, and health-related social support [7,12,13]. However, electronic health tools provide little value if the intended actors of health care systems, such as patients and health professionals, lack the skills to engage them effectively. This makes the skills to search, select, appraise, and apply web-based health information and health care–related digital apps increasingly important in the health care area [5,14,15]. These skills are called digital health literacy or eHealth literacy [16-18].

Digital health literacy is a congregate set of 6 basic skills (traditional literacy, health literacy, information literacy, scientific literacy, media literacy, and computer literacy) [15]. On the other hand, digital health literacy not only requires the ability to search for health-related information, understand the information, and apply it appropriately but also indicates advanced technology that involve patient empowerment and involvement, sharing information, and social networking [19,20]. In the context of digitization, it needs to be emphasized that users are not just passive recipients but rather active participants in the communication process by interacting with existing content or by sharing their own health-related information [21]. In this regard, health care professionals should be able to identify and use reliable health care information sources from the internet and other relevant sources of information to make evidence-based medical decisions as well as to improve health care service delivery [10,15,22,23].

Ethiopia is at a pivotal moment in its efforts to strengthen the health status of its population. As Ethiopia has made progress in reaching the health-related Millennium Development Goals, the government has realized that these advances need to be accelerated if targets in the areas of maternal and child mortality and infectious diseases are to be achieved.

Even the interaction between technology and health care has a long history, as the embracing of digital health is slow because of limited infrastructural arrangements, capacity, and political willingness [7]. Regardless of the escalating number of internet users and mobile phone penetration around the globe, the implementation of digital health systems continues to be challenging, especially in resource-limited countries [24-26]. Ethiopia is in the process of putting in place a digital health program to improve the delivery of health care services. In line with this, the Ethiopian government has implemented a strategy that focuses on digitalizing the health system [27-30]. However, the low internet penetration in Ethiopia—less than 2%, [31]—and the skills needed to find and evaluate web-based resources remain a challenge for the sustainability of digital health programs [32].

Literature has depicted that digital health literacy positively influences health-promoting behaviors and people’s health-related quality of life. Digital health literacy is also influenced by educational background, motivation for seeking the information, the technologies used, frequent internet use, computer literacy, digital health training, knowledge regarding the availability and importance of health information, perceived usefulness, having higher internet efficacy, and attitude toward using web-based health information resources [32-40]. Most of the previous studies conducted in Ethiopia did not examine the potential factors of digital health literacy skill. Some of them focused on digital health strategies, web-based health information source, and the application of ICT and use of computer in the health care area.

Addressing these problems will have a practical benefit for improving the quality of health and health care services. Moreover, evaluating health professionals’ digital health literacy skills would allow the government to identify a variety of literacy levels and hindering factors to generate a proper response accordingly. Therefore, this study was aimed at assessing the digital health literacy skill and associated factors among health professionals working at public health facilities in the Illubabor and Buno Bedele zones, Ethiopia.

## Methods

### Study Design and Setting

The cross-sectional study was conducted from January to April 2021 at selected public hospitals in the Illubabor and Buno Bedele zones, Oromia Regional state, Ethiopia. Currently, the Illubabor zone has 14 woredas and 1 administrative town. as well as 41 health centers and 2 hospitals (1 referral hospital and 1 primary hospital). Mettu Karl Referral Hospital and Darimu Hospital provide primary and advanced health care service for the Illubabor zone. The Buno Bedele zone has 10 woredas and 1 administrative town, as well as 3 hospitals called Buno Bedele General Hospital, Dambi Hospital, and Chora Hospital. The health systems of both zones include hospitals, health centers, and health posts.

### Population

The source population were all health professionals working at the public hospitals of the 2 zones [41]. All selected health professionals working at the public health hospitals of the 2 zones and available during data collection time were included in the study. Health professionals who have less than 6 months working experience from the 2 zones were excluded from this study [41].

### Sample Size and Sampling Procedure

The sample size was calculated using a single population proportion formula by considering the following assumptions.



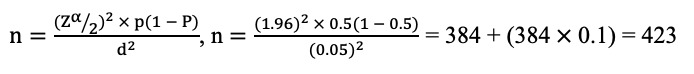



Therefore, 423 participants were included for this study.

### Data Collection and Data Quality Control

The design and development of the self-administered structured questionnaire for this study was guided by literature reviews. Questions were adapted from other studies [17,33,39,42-45]. The questionnaires gathered information about the participants’ sociodemographic characteristics, computer skills, attitude, access, and technology-related factors. Data were collected using a self-administered questionnaire that was prepared in English. A total of 4 degree-holding health professionals and 8 health professionals participated in the data collection process as supervisors and data collectors, respectively.

To ensure the quality of data, a pretest was conducted at Jimma University, which has a similar population to our study setting, by taking 10% of respondents from the total sample size. Subsequently, the necessary correction was completed based on the pretest finding. The validity of the questionnaire was determined based on the view of experts, and the reliability was obtained by calculating the Cronbach α (.7) [41]. The scale evidenced high internal consistency (overall Cronbach α=.87). Data collecting material was checked for spelling errors and its completeness and code before the actual data collecting date.

The data were also checked daily by the supervisor and the investigator for its consistency and completeness.

A 2-day training was given to data collectors about the purpose of the study, the content of the questionnaires, and all the study protocols to be followed throughout the data collection. Health facilities were assigned to each data collector so as to increase the response rate. Supervisors conducted regular supervision. Data backup activities such as storing data in different places and duplicating hard and soft copies of data were performed to prevent data loss. Before running the logistic regression model, assumptions were checked for outliers, multicollinearity, and independent error terms. Multicollinearity was tested by running a false linear regression iterating the independent variables as the independent variable, and the result showed the entire variance inflation factor value as less than 3 and tolerance as greater than 0.7, which demonstrated the absence of multicollinearity [41]. The data were also checked for outliers by a box plot, and no outshining outlier effect was observed. The model’s goodness of fit was also checked.

We used omnibus tests of model coefficients for the overall (global) fitness of the model and a Hosmer-Lemeshow test for the fitness of the data to the model. Consequently, the omnibus test result was significant with a *P* value <.05, and the Hosmer-Lemeshow test showed a good model fit with a *P* value of .61.

### Data Management and Analysis

The data was entered using Epi Info (version 7; Centers for Disease Control and Prevention), and analysis was done using SPSS (version 20; IBM Corp) software. Frequency and descriptive statistics were used to describe respondents’ characteristics.

Binary logistic regression analysis was conducted to assess the effect of selected variables on digital health literacy skill. Variables having a *P* value <.2 on the bivariate analysis were entered into a multivariable logistic regression analysis to check for confounding effects on the association from bivariate analysis. The strength of association was described at 95% CI, and a *P* value <.05 was considered significant. Odds ratios were used to determine the strength of association. Multicollinearity was checked between independent variables.

### Ethics Approval

All methods of the study were carried out in accordance with relevant guidelines and regulations. All experimental protocols were approved by the ethical review board of Mettu University (approval ARCSV/161/2013). A permission letter was received from each hospital. After the objective of the study was explained, informed consent was obtained from all study participants. Moreover, privacy and the confidentiality of information were strictly guaranteed by all data collectors and investigators. The information retrieved was used only for the study. Thus, the names of participants and other personal identifiers were not included in the data collection tool.

### Result

#### Participants

In total, 401 study subjects were included in the study. The response rate was 94.8% (401/423). The mean age of the participants was 32.13 (SD 11.2) years. Of the 401 participants, 217 (54.1%) were aged <30 years and 248 (61.8%) were male. Almost half (n=206, 51.4%) of the participants had a monthly income of <5000 Ethiopian birr (<US $94.56), and only 35 (8.7%) had a monthly income of 10,000-15,000 Ethiopian birr (US $189.12-283.68). Regarding education, 119 (54.6%) participants had a diploma and only 46 (11.5%) had a master’s degree and above. Additionally, 211 (52.6%) health professionals had <5 years of working experience and only 48 (12%) had >10 years of working experience. Among the participants, 124 (30.9%) were nurses and 107 (26.7) were physicians, as shown in Table 1.

**Table 1:** Sociodemographic characteristics of health professionals.

Variable, category			Participant (N=401), n (%)
**Age (years)**			
	<30	217 (54.1)	
	30-39	94 (23.4)	
	40-49	49 (12.2)	
	>49	41 (10.2)	
**Gender**			
	Female	153 (38.2)	
	Male	248 (61.8)	
**Monthly income (Ethiopian birr)**			
	<5000 (<US $94.56)	206 (51.4)	
	5000-10,000 (US $94.56-189.12)	114 (28.4)	
	10,000-15,000 (US $189.12-283.68)	35 (8.7)	
	>15,000 (>US $283.68)	46 (11.5)	
**Educational status**			
	Diploma	219 (54.6)	
	Bachelor’s degree	136 (33.9)	
	Master’s degree and above	46 (11.5)	
**Experience (years)**			
	<5	211 (52.6)	
	5-10	142 (35.4)	
	>10	48 (12)	
**Professional category**			
	Nurse	124 (30.9)	
	Physician	107 (26.7)	
	Midwifery	98 (24.4)	
	Laboratorian	49 (12.2)	
	Others	23 (5.7)	

#### Digital Health Literacy Level

The median digital health literacy score was 27.4 (SD 8.3). Scores less than the median value were labeled as low digital health literacy level, and scores greater than or equal to the median value were labeled as high digital health literacy level. From the total, 43.6% (175/401; 95% CI: 40.7-54.12) had high digital health literacy skills during the pandemic. Associated factors with a *P* value <.2 from the bivariate analysis were included in the final multivariable logistic regression model to control the effect of confounding as shown in Table 2.

**Table 2:** Digital health literacy level questions among health professionals (N=401).

Items			Strongly disagree, n (%)		Disagree, n (%)		Neutral, n (%)		Agree, n (%)		Strongly agree, n (%)
**Digital health literacy skills**											
	The internet is useful in helping you make decisions about your health	123 (30.7)		113 (28.2)		48 (12)		67 (16.7)		50 (12.5)	
	The internet is important for you to be able to access health resources	121 (30.2)		115 (28.7)		45 (11.2)		71 (17.7)		49 (12.2)	
	I know what COVID-19–related health resources are available on the internet	43 (10.8)		55 (13.8)		81 (20.2)		121 (30)		101 (25.2)	
	I know where to find helpful health resources on the internet	41 (10.2)		47 (11.7)		91 (22.7)		119 (29.7)		103 (25.7)	
	I know how to find helpful COVID-19 pandemic resources on the internet	6 (1.4)		56 (14)		78 (19.5)		181 (45.1)		80 (20)	
	I know how to use the internet to answer my questions about the COVID-19 pandemic	4 (1)		52 (13)		75 (18.7)		179 (44.6)		91 (22.7)	
	I know how to use the health information about the COVID-19 pandemic I find on the internet to help me	8 (2)		54 (13.5)		70 (17.4)		175 (43.6)		94 (23.5)	
	I have the skills I need to evaluate the COVID-19–related resources I find on the internet	11 (2.7)		48 (12)		69 (17.2)		172 (42.9)		101 (25.2)	
	I feel confident in using information from the internet to make COVID-19–related decisions	8 (2)		70 (17.5)		54 (13.5)		147 (36.6)		122 (30.4)	

#### Internet Use

Overall, of the 401 respondents, 49.3% (n=198) reported that they used the internet and 203 (50.6%) reported that they have never used the internet. Of the 198 internet users, about one-half (n=99, 50%) accessed the internet or email on a daily basis. Most (304/401, 75.8%) health professionals had access to the internet from home.

#### Factors Associated With Digital Health Literacy Level

All variables were entered into the binary logistic regression model. Computer literacy, marital status, educational status, monthly income, place of residence, self-efficacy, perceived ease of use, perceived usefulness, attitude and knowledge, and the frequency of internet use were significant factors associated with digital health literacy from the bivariable analysis. All variables were entered into the multivariable logistic regression model. Computer literacy, educational status, monthly income, place of residence, self-efficacy, perceived ease of use, perceived usefulness, attitude and knowledge, and the frequency of internet use were significant factors associated with eHealth literacy from the multivariable analysis. Accordingly, those having high computer literacy were 4.43 (95% CI 2.34-5.67; *P*=.01) times more likely to have a high eHealth literacy level than those who have low computer literacy. Similarly, respondents who have a master’s degree and above were 3.42 (95% CI 2.31-4.90; *P*=.02) times more likely to have a high eHealth literacy level than those who have a bachelor’s degree or diploma. Health professionals who used the internet daily were 4.00 (95% CI 1.78-4.02; *P*=.03) times more likely have a high eHealth literacy level than those who used less than 1 day per week. Similarly, respondents who perceived eHealth as being easy to use were about 2.65 (95% CI 1.35-4.65; *P*=.04) times more likely to have a high eHealth literacy level than respondents who perceived eHealth as not being easy to use. Respondents who earn a monthly income of >15,000 Ethiopian birr (>US $283.68) were 7.55 (95% CI 6.43-9.44; *P*<.001) times more likely to have a high eHealth literacy level than respondents who received income of <15,000 Ethiopian birr (<US $283.68). Those who have good knowledge of eHealth were 2.22 (95% CI 1.32-4.03; *P*=.04) times more likely to have a high eHealth literacy level than respondents with low knowledge of eHealth. Attitude was also found to be a significant factor that affected the level of eHealth literacy; respondents with favorable attitudes about eHealth were about 3.11 (95% CI 2.11-4.32; *P*=.04) times more likely to have a high level of eHealth literacy than health professionals who had unfavorable attitude toward eHealth. Additionally, health professionals who perceived usefulness were about 3.43 (95% CI 2.43-5.44; *P*=.02) times more likely to have a high eHealth literacy level than respondents who did not perceived usefulness, as shown in Table 3.

**Table 3:** Bivariable and multivariable analysis of factors associated with digital health literacy among health professionals.

Variables		Digital literacy level, n		COR^a^ (95% CI)	AOR^b^ (95% CI)	*P* value
		High	Low			
**Computer literacy**						
	High computer literacy	84	88	5.10 (2.43-7.65)	4.43 (2.34-5.67)	.01
	Low computer literacy	190	39	1	1	
**Marital status**						
	Married	211	98	1.40 (1.12-2.99)	1.21 (0.98-2.31)	.07
	Not married	69	23	1	1	
**Educational Status**						
	Diploma	145	74	1	1	
	Bachelor’s degree	101	35	1.50 (1.21-3.4)	1.49 (0.97-2.54)	.11
	Master’s degree and above	41	5	4.18 (2.51-6.54)	3.42 (2.31-4.90)	.02
**Monthly income (Ethiopian birr)**						
	<5000 (<US $94.56)	50	156	1	1	
	5000-10,000 (US $94.56-189.12)	56	58	3.01 (2.11-5.04)	1.90 (0.96-4.11)	.09
	10,000-15,000 (US $189.12-283.68)	19	16	3.70 (3.21-5.03)	2.96 (2.55-4.04)	.01
	>15,000 (>US $283.68)	34	12	8.84 (5.44-11.65)	7.55 (6.43-9.44)	<.001
**Frequency of internet use**						
	Less than 1 day per week	55	101	1	1	
	Several days per week	67	48	2.56 (1.89-3.94)	2.31 (1.76-3.88)	.03
	Daily	76	54	2.58 (1.81-3.81)	4.00 (1.78-4.02)	.03
**Knowledge**						
	Good knowledge	190	56	2.39 (1.51-4.80)	2.22 (1.32-4.03)	.04
	Poor knowledge	91	64	1	1	
**Attitude**						
	Favorable attitude	210	56	3.38 (2.41-4.80)	3.11 (2.11-4.32)	.04
	Unfavorable attitude	71	64	1	1	
**Perceived ease of use**						
	Easy	147	80	2.73 (1.51-4.74)	2.65 (1.35-4.65)	.04
	Not easy	70	104	1	1	
**Perceived usefulness**						
	Useful	158	69	4.35 (2.76-6.89)	3.43 (2.43-5.44)	.02
	Not useful	60	114	1	1	

^a^COR: crude odds ratio.

^b^AOR: adjusted odds ratio.

## Discussion

### Principal Findings

This study attempted to describe and assess the digital health literacy of health professionals and significant factors. Digital health literacy is the major barrier to access updated health information for health professionals, specifically during public health emergencies. Overall, the findings from this study suggested that the digital health literacy level was low (43.6%; 95% CI 40.7-54.12), which was consistent with previous findings [32,38,46-48]. At the same time, our result was lower than the study findings in the Netherlands (76%) [17], Pakistan (54.3%) [42], and Iran (54.4%) [48]. Likewise, a study in Chicago reported that one-quarter of health professionals had low digital health literacy [49]. This variation could be due to the fact that our study was focused on the resource-limited country setting of Ethiopia, in which the internet penetration was very low. Surprisingly, our finding was lower than those of the studies conducted in Northwestern Ethiopia, which reported that 60% [36] and 69.3% [50] of respondents possessed high digital literacy. This could be due to infrastructure differences in the selected health care facilities.

In contrast, our finding was higher than that of the study conducted in Korea, where digital health literacy was 38.8% [51]. These different findings may be related to the difference between the target populations of these studies. In this study, the participants were health professionals, whereas the study conducted in Korea was among nursing students.

Digital health literacy level is interlinked with sociodemographic, behavioral, and technological factors. Our finding implies that the computer literacy level of health professionals had a direct relationship with digital health literacy level. The professionals who had a high computer literacy level were 4.43 (95% CI 2.34-5.67) times more likely to have higher digital health literacy, which was supported by a previous study [52]. This finding was due to the fact that computer literacy, which is the knowledge and ability to use computer-related technology, made the interaction of health professionals with digital health applications easier.

Professionals who had a master’s degree were 3.42 (95% CI 2.31-4.90) times more likely to have a high level of digital health literacy than health professionals who were only diploma holders. This finding is supported by studies conducted elsewhere [38,45,53] and could be due to the fact that higher education makes one more proficient with digital tools use and web-based resources. This finding strengthens the concept that higher education is interlinked with higher use of the internet for health purposes [54].

Similar to previous finding elsewhere [33,38,50,55,56], this study revealed that health professionals who had a higher monthly income were more likely to have a high digital health literacy level, which was 2.96 (95% CI 2.55-4.04) and 7.55 (95% CI 6.43-9.44) times higher for health professionals who had a monthly income of 10,000-15,000 Ethiopian birr (US $189.12-283.68) and >15,000 Ethiopian birr (>US $283.68) than health professionals who earned <5,000 Ethiopian birr (<US $94.56), respectively. This finding might be due to high-earning health professionals having the necessary digital tools such as computer, smart phone, and tablets. However, this study was in contrast with a previous study in Northwest Ethiopia, which reported that a higher monthly income lowers digital health literacy level [32]. This difference might be due to the study setting and participants’ sociodemographic characteristics.

Health professionals who used the internet daily were 4.00 (95% CI 1.78-4.02) times more likely to have digital health competency than those who did not use the internet at least one day per week. This finding is in line with previous studies conducted in different areas [38,49,52,57,58] and could be due to the fact that the internet is the precondition for using digital health tools.

The result of this study indicates that health professionals who were knowledgeable on health information sources were about 2.22 (95% CI 1.32-4.03) times more likely to have higher digital health literacy than who had poor knowledge, and this result was supported by previous studies [50,59,60]. The possible explanation for this finding could be that digital health–related knowledge builds the competency and skill for using web-based health information sources, and knowledgeable health professionals can look up what and how to do a skill or task.

Health professionals who had a favorable attitude were 3.11 (95% CI: 2.11, 4.32) times more likely to have higher digital health literacy. This result was consistent with previous studies [37,38,50,60]. The explanation for this result could be that the attitude of health professionals helps them be more committed, since they do not consider it to be wasting their time when using digital health tools. Having a favorable attitude indicates an understanding of the relevance and use of digital health tools that could lead to a high literacy level by creating motivated health professionals. Moreover, the change in attitude might lead the overall technological and cultural change.

Regarding the perceived ease of use, this study implied that health professionals who perceived using digital health tools as being easy were 2.65 (95% CI 1.35-4.65) times more likely to have a higher digital health literacy level than their counterparts. This finding could be due to the fact that health professionals who consider using digital tools as being easy were more confident when practicing and building their literacy, and it is known that the perceived ease of use could influence health professionals’ acceptance of digital health information technologies [61].

Health professionals who perceive digital tools as useful were 3.43 (95% CI 2.43-5.44) times higher in digital health literacy. This finding is in line with a previous study in Northwest Ethiopia [36] and might be due to the perceived benefit from using digital health tools that enhanced health professionals’ attitude, which ultimately leads to sustainably practicing the use of the tools.

### Limitations

First, the study was a facility-based cross-sectional study, which could not be used to identify causal inference. Second, the study was conducted at health facilities and might not be generalizable to all administrations of the country. In addition, the study was not able to include health professionals working at private health facilities. Finally, we recommend repeating our study in different parts of the country to determine the level of eHealth literacy, including health professionals from private hospitals.

### Conclusions

In general, less than half of the study participants had a high digital health literacy level. High computer literacy, master’s degree and above, frequent internet use, perceived ease of use, monthly income of >15,000 Ethiopian birr (>US $283.68), good knowledge of digital health, favorable attitudes, and perceived usefulness were the most determinant factors associated with digital health literacy skills. Having a high computer literacy, frequent use of internet, perceived ease of use, perceived usefulness, favorable attitude, and high level of education will help promote the level of digital health literacy. However, the level of digital health literacy among health professionals in this study area was relatively low. Thus, an attempt needs to be taken to fill the gap in digital health literacy among health professionals that will help them increase their productivity and increase the relevance of digital health to their day-to-day tasks.

## Abbreviations

AOR: adjusted odds ratio
ICT: information and communication technology

## Data Availability

The data sets generated and/or analyzed during the current study will be available upon reasonable request from the corresponding author.
